# Tracking the Prevalence of Depression Among Older Adults in Singapore: Results From the Second Wave of the Well-Being of Singapore Elderly Study

**DOI:** 10.1155/da/9071391

**Published:** 2025-04-16

**Authors:** P. V. AshaRani, Edimansyah Abdin, Kumarasan Roystonn, Fiona Devi, Peizhi Wang, Saleha Shafie, Vathsala Sagayadevan, Anitha Jeyagurunathan, Boon Yiang Chua, Bernard Tan, Janhavi Ajit Vaingankar, Fengyuan Yao, Harish Magadi, Stefan Ma, Wai Leng Chow, Paul McRone, Martin Prince, Rathi Mahendran, Li Ling Ng, Siow Ann Chong, Mythily Subramaniam

**Affiliations:** ^1^Research Division, Institute of Mental Health, Singapore, Singapore; ^2^Geriatric Psychiatry, Institute of Mental Health, Singapore, Singapore; ^3^Epidemiology and Disease Control Division, Ministry of Health, Singapore, Singapore; ^4^Disease Policy and Strategy, Ministry of Health, Singapore, Singapore; ^5^School of Health Science, University of Greenwich, London, UK; ^6^Health Service and Population Research Department, Section of Epidemiology, King's College London Institute of Psychiatry, London, UK; ^7^Mind Care Clinic @ SBF, Farrer Park Hospital, Singapore, Singapore; ^8^Department of Psychological Medicine, Changi General Hospital, Singapore, Singapore

**Keywords:** depression, epidemiology, older adults, prevalence

## Abstract

**Background:** Late-life depression has serious public health implications due to its impact on healthcare systems and the economy. As the prevalence of depression tends to change over time across populations, continuous disease surveillance is warranted to inform evidence-based preventive interventions. The well-being of the Singapore elderly (WiSE) is the second study in the series that looked at the prevalence and correlates of depression in a multiethnic population in Singapore.

**Methods:** This single-phase and comprehensive cross-sectional study employed stage 1 diagnosis of geriatric mental state-automated geriatric examination for computer-assisted taxonomy (GMS-AGECAT) to capture depression and subsyndromal depression.

**Results:** The prevalence of depression and subsyndromal depression was 4.4% and 11.9%, respectively, compared to 3.7% and 13.4% in 2013 (*p*=0.425). Indians, those who were divorced and had below primary education had higher odds of depression (2.2, 3.6, and 4.2 times, respectively). Depression was associated with severe disability, loneliness, suicidal ideations, poor life satisfaction, health status, and social connections.

**Conclusion:** Despite a decade of preventive efforts for the population, there has not been any decrease in the prevalence of depression. There needs to be continued efforts to strengthen prevention, detection, and access to care of those with depression. A multiprong community–based preventive strategy focusing on social as well as health factors is needed to promote social connections, reduce loneliness, and promote the overall wellbeing of the elderly.

## 1. Introduction

Depression is one of the most common psychiatric disorders, with a global prevalence of 5% among adults [1]. Depression leads to reduced psychosocial functioning, health-related quality of life, and higher mortality, and is thus an economic burden for the individual and the society [2–4]. Nearly 31% of the patients with depression exhibit treatment resistance in the US, which is associated with higher treatment costs, unemployment, and healthcare burden [5]. According to the World Health Organization (WHO), depression is a leading cause of disability and ill health [6], and around 50% of people with depression remains untreated, even in high-income countries. The WHO has urged countries to take action, emphasizing a higher risk of noncommunicable diseases (such as diabetes, substance abuse, and heart disease) among individuals with depression.

The world is facing a demographic shift with its rapidly aging population. WHO estimates that nearly one in six people will be over the age of 60 by 2030 [7]. Depression among older adults has a different phenomenology than early-life depression, characterized by higher somatic symptoms, greater disease severity, more agitation, and less guilt [8, 9]. Various factors such as loneliness and health status have been associated with depression [10, 11]. Persistent depression among older adults is also associated with a higher risk of dementia [12]. Thus, it is clear that later-life depression has grave healthcare and economic implications. A systematic review and meta-analysis of 42 studies showed a global prevalence of 31.7% of depression in older adults [13]. Another recent systematic review of 55 studies showed an overall prevalence of 35.1% in older adults [14]. The prevalence of depression was higher among older adults from developing nations (40.8%) than in developed nations (17.1%) [13]. Also, the prevalence of depression was significantly higher among older adults in urban areas than in rural areas in developed nations, but not in developing nations [15].

Studies have monitored the prevalence of depression over time and showed mixed results across countries, which can be attributed to methodological differences or population characteristics. A comparison of data from population-level cohorts in the UK showed an increase in antidepressant use across two decades (years 1993 and 2011), but no increase in overall prevalence of depression [16]. Contrary to this, a Danish study showed an increase in the prevalence of depression from 2% to 4.9% between 2000 and 2006. This increase was observable among women (>40 years) and those from lower socioeconomic status [17]. Another cohort study conducted over a 11-year period in Norway showed an increase in the prevalence of depression along with an incidence of 10% in all age cohorts aged 81 years and older at follow-up [18]. Another systematic review of 81 studies showed an increasing trend in the prevalence of depression in China among older adults from 1990 to 2000 and a decrease thereafter until 2010 [19]. The prevalence of depression increased with age, with a higher increase (9.6%) noted in those aged 86–90 years at follow-up [19]. A study conducted in India among community-dwelling older adults showed a high prevalence of depression (67.5%) [20] compared to other countries. The study also showed that those who were aged 80 and above, females, widowed, living alone, and having no social or family support had a higher risk of developing depression. There has been an increasing interest in subsyndromal depression over the past few decades. Research on subsyndromal depression among older adults has yielded varying prevalence rates across different populations. A study in the United States reported a prevalence of 13.8% among older adults, with participants showing higher odds of lifetime and new-onset mood disorders [21]. In contrast, a study conducted in China found a significantly higher point prevalence of 38.7% in community-dwelling older adults [22]. A meta-analysis of 77 studies revealed an overall prevalence of 18.6% for subsyndromal depression in older adults, noting that rates varied across continents and depending on the assessment tools used [23].

Oh et al. [24] identified several factors associated with subsyndromal depression, including gender, socioeconomic status, social support, and sleep quality. Importantly, individuals with subsyndromal depression face an increased risk of developing clinical depression later in life [25]. The prevalence of depression and subsyndromal depression among older adults varies across time, the socioeconomic status of the country and population, and geographical areas. Therefore, in view of these variations, it is imperative to do population-specific surveillance from time to time to understand the change in depression trends over time. Such disease surveillance forms the cornerstone for preventive efforts and timely community-level interventions.

Singapore, a multiethnic island nation in Southeast Asia, has a population of 5.92 million. Those who are 60 years of age and older constitute more than one million (~17.4%) [26] of Singapore's total population. This demographic shift has an immense socioeconomic impact, with a lower labor force, a higher disease burden, and thus, higher healthcare utilization. The well-being of the Singapore elderly (WiSE) study conducted during 2012–2015 (WiSE 1 study) showed a prevalence of 3.7% and 13.4% of depression and subsyndromal depression using the geriatric mental state-automated geriatric examination for computer-assisted taxonomy (GMS-AGECAT). Those with depression had poorer life satisfaction, more disabilities, and more comorbidities [27]. The Ministry of Health (MOH) in Singapore has strengthened community-level preventive efforts to detect and treat depression since then. MOH, together with the Ministerial Committee on Aging (MCA), implemented an action plan for successful aging in 2015, which translated to 60 community level programs in collaboration with various community-level partners to promote healthy aging, focusing on areas such as social inclusion, health, housing, transport, the elderly, healthcare, et cetera, working together with agencies and community outreach teams [28]. Moreover, the silver generation ambassadors (community volunteers who attend to older adults to connect them to relevant policies or programmes supporting active aging) and the Health Promotion Board (HPB) organize workshops and home visits to support the elderly who are socially isolated to reduce their risk of developing of mental illnesses [29]. The current study is the second wave of the WiSE study, which aimed to understand the prevalence of depression and subsyndromal depression, monitor the changing patterns in prevalence, and understand the correlates of depression in older adults in Singapore. The current study will shed light on the change in the prevalence of depression, thereby, facilitating data-driven healthcare and community resource planning.

## 2. Methods

### 2.1. Sampling and Sample Size

The second WiSE study was a cross-sectional nationwide survey that examines the prevalence of dementia and depression among older adults in Singapore. The study followed the same methodology as the previous study [27, 30]. Briefly, study data was collected between March 2022 and September 2023 through face-to-face interviews. The sample size was estimated based on a prevalence rate of 10% for dementia as established in the previous WiSE study [27]. Assuming a statistical power of 0.80, type 1 error of 0.05, and a margin of error of 2% for overall prevalence, a sample size of 2000 was estimated to be required for the study. Survey weights were employed during analysis to account for nonresponse and disproportionate stratified sampling.

### 2.2. Participants

Participants who were 60 years of age or older were randomly selected from a national-level administrative database containing the household addresses of all Singapore residents. A stratified random sampling was employed, where participants were sampled disproportionately from three different strata: age (60–74 years; 75–84 years; >85 years), gender, and ethnicity (Chinese, Indian, Malay, and others). Older adults who were not living in Singapore during the active survey period or were institutionalized (e.g., in prison; those in nursing homes were included) were excluded from the study. All participants received a complete 10/66 assessment [27].

### 2.3. Procedures

Interviewers were trained to administer the survey questionnaires and assessments through a 2-week training program that included face-to-face hands-on training, round-robins, learning videos, and interactive sessions by clinicians. All the interviewers underwent an evaluation by the trained study team members, and those who were successful, were further observed in the field by a study team member to identify and correct any challenges or errors that happened during the field work. The contact records were checked regularly as a part of quality control and queried if any discrepancies were spotted. Additionally, 20% of the interviews done by each of the interviewers were subjected to quality checks through phone or door-to-door visits, where part of the questionnaires was administered to capture any discrepancies.

An invitation letter was sent to all the households at least 1 week before the actual visit by the interviewer to invite the participant, notify the participant and family members about the purpose of the study, and provide additional information on the study. The interview was conducted face-to-face through computer-assisted personal interviewing. The interview lasted for 2–3 h for each older adult. The older adult was recruited together with an informant in a 1:1 dyad, except in cases where the older adult was living alone and was unwilling to identify anyone as an informant or the informant selected by the older adult refused to participate. An informant was defined as someone who was involved in most of the care decisions of the older adult and did not include paid caregivers. Written informed consent was obtained from all the participants and from their legal representative if the participant was cognitively incapable of giving consent. The study procedures were approved by relevant ethics committees (National Healthcare Group Domain Specific Review Board (DSRB) and SingHealth Centralized Institutional Review Board (CIRB) Ref: 2020/01400). The response rate of the survey was 62.7%.

### 2.4. Assessments

#### 2.4.1. Sociodemographic Factors

Participants' age, gender, ethnicity, education, marital status, income, and employment status were collected using a structured questionnaire.

#### 2.4.2. The GMS

As described in the previous WiSE study [27], the current study also employed the stage 1 GMS-AGECAT depression syndrome module.

The GMS assessment captures responses through a semistructured interview and a rating scale that considers psychopathology, sensory functions, and frailty. The GMS-AGECAT system generates four syndrome clusters based on the input: organicity, schizophrenia and related paranoia, depression, and anxiety neurosis. Each syndrome is classified according to severity levels ranging from 0 to 5, where 0 indicates no symptoms and 5 represents very severe symptoms. Stage 1 diagnosis captures severity levels 3 and above (marked as severely affected and very severely affected) as depression (cases), while levels 1 and 2 (subcases) are classified as subsyndromal depression.

#### 2.4.3. Suicidality

Suicidality was measured using a single item [31]: “Have you ever felt that you would rather be dead because life has become a burden for you?” Suicidal attempts were measured using a follow-up question: “Did you actually try anything?” Response options included “no (0),” “yes (1; mild-to moderate),” “yes (2; severe, frequent or persistent).” Responses 1 and 2 were recorded as “yes,” while response 0 was recorded as “no.”

#### 2.4.4. Disability

Disability was captured using the WHO Disability Assessment Schedule 2.0 (WHODAS 2.0) [32]. WHODAS covers six domains of functioning: cognition, mobility, self-care, getting along, life activities, and participation in activities. It is scored on a five-point scale (0–4), where 0 means no difficulty and 4 indicates extreme difficulty. A total score was obtained by the summation of the domain scores. Those who reported 15 days or more of disability days were classified as severe disability [33].

#### 2.4.5. Other Questionnaires From the 10/66 Dementia Research Group's Assessment Tool Kit [31]

##### 2.4.5.1. Overall Health Status

Overall health status was captured using the question, “How would you rate your overall health in the past 30 days?” The responses varied from 0 to 5, where 0 indicated very good and 5 indicated very bad. Original responses 0 and 1 were recorded as 1 (good and very good), while other responses were recorded as 0 (moderate to very bad).

##### 2.4.5.2. Other Health Conditions

This was captured by asking the participant if a doctor has ever diagnosed them with any of the following conditions: hypertension (HBP), heart problems, stroke, diabetes, or transient ischemic attacks (TIAs). Other physical health conditions were captured through self-reports (arthritis or rheumatism, eyesight problems, hearing difficulties, persistent cough, asthma, stomach or intestinal problems, faints or blackouts, paralysis, weakness or loss of limbs, skin disorders, and cancers). The participants were asked if they had any of the above physical conditions that interfered with their activities. The responses were captured on a four-point scale (0 = does not have a health problem, 1 = has a problem, interferes not at all, 2 = has a problem, interferes a little, and 3 = has a problem, interferes a lot). Responses 1–3 were recorded as 1 (yes), while 0 was captured as 0 (no).

##### 2.4.5.3. Health Status, Physical Activity, and Satisfaction With Life

Participants' overall health status was assessed through a single item: “How would you rate your overall health in the past 30 days?” The responses were captured on a five-point scale (4 is very bad, 3 is bad, 2 is moderate, 1 is good, and 0 is very good). Physical activity was captured by asking, “Taking into account both work and leisure, would you say that you are “very physically active,” “fairly physically active,” “not very physically active,” or “not at all physically active?” Responses “very physically active” and “fairly physically active” were combined and recorded as “very physically active and fairly physically active,” while all others were recorded as “not very physically active or not at all physically active.” Satisfaction with life in general was assessed by asking, “How would you describe your satisfaction with life in general at the present time?” The response options included “good,” “fair,” or “poor.” Responses “good” and “fair” were recorded as “satisfied,” while “poor” was recorded as “dissatisfied.”

##### 2.4.5.4. Social Support and Loneliness

Social support was measured using a questionnaire that asked, (i) “How often do you see any of your children or other relatives to speak to?” (ii) “How often do you see any of your neighbors to have a chat or do something with?” (iii) “All in all, are you satisfied or dissatisfied with the help and support you can get from your close friends?” The response options varied from “never,” “less than monthly,” and “monthly,” “daily,” “2–3 times a week,” and “weekly” for questions (i) and (ii). For question (iii), the response options were “satisfied” or “dissatisfied.”

Loneliness was captured using two items: “Do you feel lonely?” and “Does it bother you very much?” The responses ranged from 1 “yes (or “abnormal”), but mild to moderate intensity, infrequent or fleeting,” 2 “yes (or “abnormal”) and severe, frequent or persistent,” and 0 “no (or “normal”).” Responses 1 or 2 that were bothered or depressed by their loneliness were recorded as “yes,” while response 0 was recorded as “no.”

### 2.5. Analysis

Statistical analyses were carried out using Stata version 17.0. A descriptive analysis was performed to describe sociodemographic profiles of the study population and establish the prevalence of depression. Multinomial logistic regression analysis was performed to examine sociodemographic correlates of depression, while a series of multivariable logistic regression and multivariable linear regression analyses were performed to examine the relationships between depression and other health outcomes where sociodemographic covariates were adjusted for eliminating confounding effects. All analyses were weighted using survey weights to ensure the survey findings were representative of the older adult population in Singapore. A statistically significant differences were evaluated at the *p*  < 0.05 level using two-sided *t* tests.

## 3. Results

### 3.1. Sociodemographic Characteristics

The sociodemographic information of the participants is included in Table 1. A total of 2010 older adults were included in the study. Nearly 53% of the sample were women and 75% were of the age range of 60–74 years. Approximately 82% were of Chinese ethnicity and 66% were married. Around 64% of the participants had a comorbid physical disorder.

### 3.2. Prevalence of Depression

The crude prevalence of depression and subsyndromal depression were 4.4% and 11.9%, respectively, compared to 3.7% and 13.4% in 2013. Whereas the age-standardized prevalence of depression and subsyndromal depression were 4.4% and 12.1%, respectively, compared to 3.7% and 13.5% in 2013. Although the prevalence of depression increased in 2023, the change was not statistically significant. A comparison of the prevalence of depression is indicated in Table 2.  Table 3 shows the prevalence of depression according to sociodemographic characteristics. A significantly higher proportion of females (5.7%) had depression compared to males (2.8%). Those who were married had a lower prevalence of depression (2.7%) than those who were never married (6.9%), widowed (7.5%), or divorced (8.9%).

### 3.3. Sociodemographic Correlates of Subsyndromal Depression and Depression

The correlates of depression and subsyndromal depression are indicated in Table 4. Those who were aged 85 years and older (OR: 2.3, 95% CI: 1.2–4.7, *p*=0.017) and females (OR: 1.9, 95% CI: 1.2–3.0, *p*=0.011) had higher odds of subsyndromal depression than those who were aged 60–74 years old and males, respectively. Those of Indian ethnicity had higher odds (OR: 2.2, 95% CI: 1.3–3.8, *p*=0.004) of having depression than those of Chinese ethnicity. Those who were divorced/separated had 3.6 times (OR: 3.6, 95% CI: 1.2–10.6, *p*=0.020) higher odds of having depression than those who were married. The odds of having depression were also significantly higher among those had below primary (OR: 4.2, 95% CI: 1.0–17.5, *p*=0.046) compared to those who had tertiary education.

### 3.4. Relationship of Depression With Other Health Conditions

Table 5 shows the prevalence and odds of other chronic conditions among those with depression. After adjusting for covariates in multivariable logistic regression analyses, individuals with arthritis or rheumatism, eyesight problems, hearing difficulty, persistent cough, asthma, stomach or intestine problems, faints or blackouts, paralysis, and skin disorders had higher odds of having depression. Those with subsyndromal depression were more likely to have higher odds of asthma, stomach or intestine problems, faints or blackouts, paralysis, and skin disorders.

### 3.5. Relationship Between Depression and Functioning, Quality of Life, and Social Support Factors

Table 6 shows the relationship between depression and disability, health status, and satisfaction with life, as well as physical activity. Severe disability and mean disability scores were significantly higher among those with depression and subsyndromal depression than those without depression. Those with depression and subsyndromal depression were less likely to endorse that their overall health status was “good and very good,” endorse their satisfaction with life as “fair or good,” and report a physical activity level as “very active and fairly active."

### 3.6. Relationship Between Depression, Loneliness, Social Support, and Suicidality

Those with depression and subsyndromal depression were satisfied with the help and support received from close friends and were more likely to endorse feeling lonely, depressed by current loneliness, and had ever felt suicidal or wished to be dead. The results are presented in Table 7.

## 4. Discussion

The current study examined the prevalence of depression and subsyndromal depression (using GMS-AGECAT) among older adults in Singapore and showed a prevalence of 4.4% and 11.9%, respectively. The prevalence of depression was not significantly higher than what was reported in the first wave of the WiSE study. A large population-level study conducted in China and Malaysia showed a prevalence of 10.5% and 11.5% for depression, respectively [34, 35]. Large epidemiological cross-sectional studies in Egypt, India, Turkey, Sri Lanka, Australia, and Japan showed a prevalence of depression ranging from 7.5% to 67.5% among older adults [14]. The prevalence observed in the current study is much lower than that of other countries. Several reviews have explored the reason for this heterogeneity. Cai et al. [14] pooled the evidence from 55 epidemiological studies and showed an overall global prevalence of 35.1% of depression among older adults. The authors also reported high variability among the results, which was attributed to the study methodology and the income levels of the countries. Another meta-analysis showed a point prevalence, 1 year and lifetime prevalence of 12.9%, 7.2%, and 10.8%, respectively, for depression in older adults from 30 countries from 1994 to 2014 [36]. The authors found that the human development index, assessment methods, number of women in the sample, and response rates were all found to be contributing to the heterogeneity in the prevalence within and across countries. Hence, a meaningful comparison of prevalence is not feasible.

We also noted an increase in the prevalence of depression between the 2013 and 2023 surveys, albeit statistically insignificant. Similar trends were noted by Tang, Jiang, and Tang [19] among older adults, who noted an increase in the prevalence of depression between 1991 and 2001 and a subsequent decrease after 2010 in China. A study conducted in the United States showed a comparable trend in the prevalence of depression, as observed in our study [37]. The authors tracked the prevalence of depression between 2005 and 2015 and showed that while the rates fluctuated, the prevalence remained close to 4% between 2005 and 2015. Hence, the increase in prevalence observed in the current study might not be of clinical significance.

The current study also identified sociodemographic groups who were at a higher risk of developing depression. Indians (vs. Chinese), those who were separated/divorced (vs. married) and those with below primary education (vs. tertiary education) had higher odds of depression. This is in line with the results from the previous WiSE study [27] and a study conducted in community-dwelling older adults in Malaysia [34]. Ethnic differences in depression have been noted previously across various ethnicities and this has been attributed to the presentation of the symptoms, cultural beliefs, social desirability, help-seeking patterns, socioeconomic status, and access to culturally or linguistically suitable care [38–40]. A study conducted in a multiethnic population consisting of workers in the UK showed that Indians had a higher risk (2.1 times) of depression than the rest [41]. The link between chronic conditions and depression among older adults is well known [42]. It is evident from the studies that older adults of Indian ethnicity have a higher prevalence of chronic conditions than Chinese, which could be one of the reasons for the higher odds of depression observed among Indians in Singapore [43].

Divorce serves as a fundamental factor contributing to numerous economic, emotional, and social hardships. It is well known that marriage improves the physical and psychological wellbeing of adults [44]. The dissolution of marriage disrupts the social life of an individual, leading to more pronounced detrimental effects on emotional relationships and financial standing. A panel study conducted in the United States demonstrated an increase in depression after divorce that peaked 4 years thereafter [45]. This was mediated through a loss of emotional support, financial hardships, and a decline in the standard of living. Large nationwide studies focusing on older adults underscored marital status as an independent predictor of depression, with those divorced having higher depression than their married counterparts, along with higher mortality rates [46, 47]. A systematic review and meta-analysis of 42 studies affirmed a strong association between divorce and depression in older adults [13]. These findings underscore the consequences of depression in divorce in older adults, emphasizing the need for interventions to support them to mitigate the challenge. We noted a higher association of various health conditions (such as asthma, skin disorders, hearing and eye problems, and intestinal problems) with depression and subsyndromal depression. Depression is one of the most common comorbidities among those suffering from chronic diseases and it is the root cause of the psychosocial burden among these patients. The underpinnings of these comorbidities are shared genetic, social, biological, and psychological factors [48, 49]. There exists a bidirectional relationship between depression and chronic conditions [49]. While depressive episodes can be precipitated by the complications and challenges faced during chronic conditions, the psychobiological changes and altered health behaviors after the diagnosis of depression can predispose people to chronic conditions [49, 50]. Managing depression among those with chronic conditions or vice versa is challenging and it leads to a higher medical and economic burden [50]. Those with asthma have a 14.1% prevalence of depression and it is associated with a higher risk of all-cause mortality among these patients [51]. Similarly, a systematic review of 77 studies evidenced that the prevalence of depression was high among those with inflammatory bowel diseases [52]. Visual and hearing problems are common in older adults as they age; however, if left untreated, they can act as chronic stressors that could trigger mood disorders [53]. Strong correlations have been noted with depression with hearing loss and visual impairment in older adults that could adversely affect their well-being [53, 54]. Therefore, healthcare professionals need to be cognizant of the risk of depression among older adults presenting with chronic conditions or other age-related physical conditions so as to provide them with necessary support and access to care.

The result from the first WiSE study also showed that those with depression tend to have severe disability, lower health status and satisfaction with life, higher loneliness and suicidality, and lower social connections. The current study corroborates findings from the previous WiSE data, which noted similar associations [27]. Those with disabilities experience five times higher mental health stress than those without [55]. Disability limits the daily functioning of an individual and can act as a strong mental health stressor in one's life, creating a feeling of “hopelessness” or “emptiness” [55]. The affected individuals may suffer from social prejudice, loss of roles, abuse, financial problems, and health inequities and are three times more likely to develop depression than the general population [56]. Noh et al. [57] studied the trajectory of depression among those with disabilities using longitudinal data from a Korean aging survey and showed that disability was a risk factor for subsequent depression and females were more likely to be affected compared to males. Life satisfaction is a known indicator of well-being among older adults that is widely used in policymaking. Life satisfaction is affected by one's purpose in life, social and family support, self-perceived health status, and the emotional support he or she receives [58]. Data from a national-level perspective cohort showed a strong correlation between higher life satisfaction and lower depression [59]. A national-level longitudinal cohort also showed that when life satisfaction increased among older adults, depression went down over a period of time [58]. The study also showed that family relationships improved life satisfaction and depression. Hence, efforts should focus on fostering strong social and family connections among older adults.

Loneliness is a risk factor for depression. The current literature shows a multifaceted relationship between loneliness and depression. Those who are depressed face social disconnectedness and functional impairments that lead to loneliness [60]. McHugh Power et al. [61], using an observational cohort, showed that prolonged depression at baseline predispose individuals to loneliness at follow-up, but not vice versa, confirming the results of Allen and Badcock [60]. However, a Spanish longitudinal study showed that chronic loneliness is a risk factor for subsequent depression [62]. Kraav et al. [63], using their population-level data, looked at the mechanism of association between depression and loneliness and reported a strong direct association between loneliness and incident depression. Although the causative factors leading to loneliness and depression remain unclear, our findings substantiate a discernible close association between loneliness and depression. Therefore, future efforts should focus on reducing loneliness among older adults to prevent depression, and thus, improve their quality of life.

We have also noted an association between suicidal ideations and depression among older adults. Suicide tends to increase during late life [64] and depression is a major contributor to it [65]. A systematic review of 35 studies conducted in older adults showed that depression is a risk factor for suicidal ideations in older adults [66]. The review also identified that the severity of depression, other comorbidities, loss of functionality, reduced social support, and loneliness can contribute to suicidality. Kin et al. [59] added to the evidence by showing through their path analysis that social support mediated the relationship between depression and suicide ideations. Given the lower likelihood of lower social connections and a higher tendency toward loneliness among the current study population, along with health-related factors, social factors should be included in the health promotion campaigns to support healthy aging and reduce the incidence of depression and suicidality.

The study has several limitations. The cross-sectional nature of the study prevents the analysis of the temporal relationships among the various variables addressed in the study. The response rate of the survey was 62.7% and those who did not participate in the study could be those who were physically or mentally ill. Thus, it is possible that the actual prevalence of depression could be higher than what is reported here. Loneliness, physical activity, and satisfaction with life were captured using a single item, not a validated scale, which is a limitation. Social desirability bias cannot be ruled out due to cultural barriers and perceptions of stigma that might interfere with disclosing mental health symptoms to the interviewer. However, the semistructured nature of the GMS made it amenable to further probing before capturing accurate data. The observed association between depression and suicidal ideation might be partially influenced by the overlap between suicidality items in the GMS-AGECAT depression assessment and our separate measure of suicidal ideation. The study was a nationwide study employing disproportionate stratified random sampling. It had a larger sample size, which was representative of the local population. Also, the assessment tools employed were well validated and were translated into all local languages. The stringent quality control and robust methodology are additional strengths of the study.

## 5. Conclusion

An increase in the prevalence of depression was noted when compared to the first phase of the WiSE study. Despite the introduction of new policies aimed at promoting mental health among the elderly, the rate of depression has not declined. This underscores the need for additional community-level initiatives grounded in evidence-based approaches. Given that loneliness increases the odds of having depression and those who are satisfied with the level of social support by friends and neighbors have a reduced OR for depression, it is crucial for partners and policy makers to work together to enhance support to seniors in the community. This collaboration is also necessary to enhance preventive measures for early screening and identification of depression, ultimately promoting timely help-seeking behaviors. Preventive strategies should be multifaceted, including social, health, and behavioral elements, to promote the mental health of the older population. Furthermore, it is important to include specific preventive efforts targeting suicide within these programmes.

## Figures and Tables

**Table 1:** Sociodemographic characteristics of the sample.

Sociodemographic characteristics	Unweighted (*n =* 2010)	Unweighted (%)		Weighted (%)	
Age group (years)					
60–74	1090	54.2		75.0	
75–84	580	28.9		18.7	
85+	340	16.9		6.3	
Gender					
Men	908	45.2		47.1	
Women	1102	54.8		52.9	
Ethnicity					
Chinese	678	33.7		81.6	
Malay	699	34.8		10.4	
Indian	616	30.6		6.4	
Others	17	0.8		1.5	
Marital status					
Never married	114	5.6		9.1	
Married/cohabiting	1168	58.2		66.2	
Widowed	617	30.8		18.7	
Divorced/separated	107	5.3		6.0	
Education status					
None	217	10.8		7.2	
Some, but did not complete primary	391	19.5		20.6	
Completed primary	568	28.4		26.5	
Completed secondary	545	27.2		28.2	
Completed tertiary	282	14.1		17.6	
Employment status					
Paid work (part-time and full-time)	633	31.6		42.2	
Unemployed	35	1.7		1.3	
Homemaker	515	25.7		16.8	
Retired	821	41.0		39.7	
Presence of any comorbid physical disorder^a^					
No	181	9		12.1	
Yes	1829	91		87.9	
Participants with informant					
No	212	10.5		13.2	
Yes	1798	89.5		86.6	
History of depression diagnosis by a doctor					
No	1976	97.9		98.1	
Yes	43	2.1		1.9	

^a^Any of the 15 health conditions.

**Table 2:** Prevalence of depression and subsyndromal depression by survey year.

Depression status	WiSE 2013			WiSE 2023			*χ* ^2^	*p* Value
	Unweighted *N*	Projected number	Weighted (%)	Unweighted *N*	Projected number	Weighted (%)		
No depression	1963	464,595	82.9	1622	809,246	83.8	0.9	0.425
Subsyndromal depression	425	75,068	13.4	270	114,801	11.9	—	—
Depression	177	20,938	3.7	118	42,089	4.4	—	—

**Table 3:** Prevalence of depression and subsyndromal depression by sociodemographic characteristics of the sample.

Variables	No depression		Subsyndromal depression		Depression	
	*n*	%	*n*	%	*n*	%
Age group (years)						
60–74	910	85.1	133	10.9	47	4.0
75–84	460	81.7	73	13.4	47	4.8
85+	252	74.4	64	18.4	24	7.2
Gender						
Men	777	88.0	96	9.2	35	2.8
Women	845	80.0	174	14.3	83	5.7
Ethnicity						
Chinese	565	84.0	83	11.8	30	4.2
Malay	579	84.2	87	12.2	33	3.7
Indian	465	77.6	97	14.9	54	7.5
Others	13	94.7	3	3.4	1	1.9
Marital status						
Never married	92	83.2	14	9.9	8	6.9
Married/cohabiting	973	85.5	148	11.8	47	2.7
Widowed	474	80.4	90	12.2	53	7.5
Divorced/separated	81	75.6	17	15.5	9	8.9
Education status						
None	162	79.4	37	14.6	18	5.9
Some, but did not complete primary	308	81.7	55	11.8	28	6.5
Completed primary	463	83.6	73	12.3	32	4.1
Completed secondary	447	84.5	65	11.1	33	4.4
Completed tertiary	237	87.0	38	11.5	7	1.6
Employment status						
Paid work (part-time and full-time)	547	87.6	63	9.6	23	2.8
Unemployed	19	75.8	11	17.5	5	6.7
Homemaker	390	79.9	89	15.6	36	4.5
Retired	663	81.6	105	12.6	53	5.8
History of depression diagnosis by a doctor						
No	1595	84.3	262	11.7	110	4.0
Yes	27	54.2	8	22.0	8	23.8

**Table 4:** Correlates of subsyndromal depression and depression.

Variables	Subsyndromal depression				Depression			
	OR	95% CI		*p* Value	OR	95% CI		*p* Value
Age group								
60–74 (Ref)	—	—	—	—	—	—	—	—
75–84	1.4	0.8	2.3	0.222	0.8	0.4	1.8	0.595
85+	2.3	1.2	4.7	**0.017**	1.0	0.3	2.9	0.935
Gender								
Men (Ref)	—	—	—	—	—	—	—	—
Women	1.9	1.2	3.0	**0.011**	1.7	0.7	3.9	0.228
Ethnicity								
Chinese (Ref)	—	—	—	—	—	—	—	—
Malay	1.0	0.7	1.5	0.919	0.9	0.5	1.7	0.719
Indian	1.4	0.9	2.0	0.093	2.2	1.3	3.8	**0.004**
Others	0.2	0.1	1.1	0.059	0.7	0.1	6.3	0.738
Marital status								
Married (Ref)	—	—	—	—	—	—	—	—
Never married	0.8	0.3	1.7	0.523	2.5	0.8	7.5	0.100
Divorced/separated	1.4	0.6	3.0	0.407	3.6	1.2	10.6	**0.020**
Widowed	0.7	0.4	1.2	0.171	2.0	0.9	4.5	0.075
Education								
Tertiary (Ref)	—	—	—	—	—	—	—	—
None	0.8	0.3	1.9	0.564	2.7	0.5	14.9	0.243
Below primary	0.9	0.5	1.8	0.784	4.2	1.0	17.5	**0.046**
Completed primary	1.0	0.5	2.0	0.929	2.7	0.6	10.9	0.174
Completed Secondary	0.9	0.5	1.7	0.773	2.7	0.7	10.3	0.138
Employment								
Employed (Ref)	—	—	—	—	—	—	—	—
Unemployed	2.2	0.9	5.6	0.084	2.1	0.6	7.4	0.261
Homemaker	1.3	0.7	2.4	0.469	1.3	0.4	3.9	0.651
Retired	1.3	0.8	2.1	0.349	2.2	1.0	4.8	0.057

*Note:* Bold values indicate statistical significance.

Abbreviation: OR, odds ratio.

**Table 5:** Prevalence and odds ratio of other health conditions in depression.

Health conditions							Multivariable logistic regression							
	No depression		Subsyndromal depression		Depression		Subsyndromal depression				Depression			
	*n*	%	*n*	%	*n*	%	OR	95% CI		*p* Value	OR	95% CI		*p* Value
HBP	934	52.5	177	59.6	83	59.4	1.3	0.9	2.0	0.225	1.2	0.6	2.2	0.600
Heart trouble	282	12.8	68	18.2	32	16.1	1.6	1.0	2.7	0.061	1.4	0.7	3.2	0.357
Stroke	103	5.9	30	9.3	12	9.2	1.5	0.7	3.1	0.249	1.3	0.5	3.9	0.601
Diabetes	552	24.9	104	28.9	48	35.3	1.2	0.8	1.8	0.460	1.6	0.8	3.1	0.194
TIAs	51	3.0	13	4.1	7	1.8	1.5	0.5	4.5	0.468	0.5	0.2	1.6	0.223
Arthritis or rheumatism	441	24.5	91	23.8	65	56.2	0.8	0.5	1.2	0.301	3.2	1.6	6.3	**0.001**
Eyesight problems	683	46.5	125	50.9	69	66.9	1.2	0.8	1.8	0.389	2.3	1.2	4.4	**0.012**
Hearing difficulty	343	17.2	83	24.5	34	31.1	1.6	0.9	2.5	0.079	2.4	1.2	4.9	**0.019**
Persistent cough	77	4.1	18	4.8	17	20.8	1.2	0.5	2.8	0.752	6.1	2.6	14.4	**≤001**
Asthma	131	5.5	40	11.1	23	17.7	2.0	1.0	4.0	**0.039**	3.3	1.4	7.7	**0.006**
Stomach or intestine problems	181	10.6	51	21.0	43	54.3	2.1	1.2	3.5	**0.007**	9.9	5.0	19.4	**≤001**
Faints or blackouts	52	4.6	19	9.6	12	27.6	2.2	1.0	4.7	**0.049**	7.2	3.1	16.8	**≤001**
Paralysis	171	5.8	62	14.1	35	27.2	2.5	1.3	4.6	**0.004**	5.1	2.3	11.0	**≤001**
Skin disorders	115	6.5	30	12.6	15	14.4	2.0	1.1	3.9	**0.034**	2.7	1.1	6.4	**0.030**
Cancer	89	5.2	17	6.3	10	8.2	1.1	0.4	2.5	0.902	1.5	0.5	4.4	0.499

*Note*: Odds ratio was derived using multivariable logistic regression after adjusting for sociodemographic variables. Bold values indicate statistical significance.

Abbreviations: HBP, high blood pressure; TIA, transient ischemic attack.

**Table 6:** Relationship between depression functioning, and satisfaction with life.

Variables	No depression		Subsyndromal depression		Depression		Subsyndromal depression					Depression				
	Mean	SD	Mean	SD	Mean	SD	*β*	95% CI			*p* Value	*β*	95% CI			*p* Value
**WHODAS 2.0 total scores**	7.4	15.9	18.1	23.4	33.2	28.5	8.6	4.9	12.2		**≤001**	23.0	15.9		30.2	**≤001**
	***n***	**%**	***n***	**%**	***n***	**%**	**OR**	**95% CI**			***p* Value**	**OR**	**95% CI**			***p* Value**

Severe disability (15 or more disability days in the past months)	215	7.1	81	23.4	49	34.2	3.5	2.0		6.0	**≤001**	5.6	2.6	12.4		**≤001**
Overall health status																
Moderate to very bad	506	31.9	140	52.0	78	74.8	Ref	—	—	—	—	Ref	—	—		
Good and very good	1110	68.1	128	48.0	39	25.2	0.5	0.3	0.7		**≤001**	0.2	0.1		0.3	**≤001**
Satisfaction with life																
Poor	7	0.5	7	4.9	17	18.4	Ref	—	—	—	—	Ref	—	—		
Fair and good	1489	99.5	238	95.1	96	81.6	0.1	0.02	0.3		**≤001**	0.019	0.005		0.1	**≤001**
Physical activity																
Not very physically active or not at all physical active	424	20.8	122	41.7	67	64.4	Ref	—	—	—	—	Ref	—	—		
Very physically active and fairly physically active	1192	79.2	146	58.3	49	35.6	0.4	0.2	0.6		**≤001**	0.1	0.1		0.3	**≤001**

*Note*: Beta coefficient (*β*) and odds ratio (OR) were derived using multivariable logistic and linear regressions after adjusting for sociodemographic variables. Bold values indicate statistical significance.

**Table 7:** Relationship between depression and loneliness, social support, and suicidality.

Variables	No depression		Subsyndromal depression		Depression		Subsyndromal depression				Depression			
	*n*	%	*n*	%	*n*	%	OR	95% CI		*p* Value	OR	95% CI		*p* Value
Feeling lonely														
No	1391	95.4	170	76.6	47	45.4	Ref	—	—	—	Ref	—	—	—
Yes	103	4.6	76	23.4	67	54.6	6.8	3.9	11.8	**≤001**	26.7	12.0	59.6	**≤001**
Depressed by current loneliness														
No	1618	99.8	253	95.3	93	84.2	Ref	—	—	—	Ref	—	—	—
Yes	4	0.2	17	4.7	25	15.8	20.8	3.7	118.7	**0.001**	105.4	13.9	799.0	**≤001**
How often do you see any of your children or other relatives to speak to?														
Never, at least monthly, or less often	126	9.3	24	8.8	12	23.6	Ref	—	—	—	Ref	—	—	—
Daily, 2 to 3 times a week or at least weekly	1326	90.7	218	91.2	93	76.4	1.0	0.4	2.1	0.944	0.3	0.1	0.9	**0.033**
Chat or do something with one of your friends	—	—	—	—	—	—	—	—	—	—	—	—	—	—
Never, at least monthly, or less often	297	26.0	66	38.4	20	33.0	Ref	—	—	—	Ref	—	—	—
Daily, 2 to 3 times a week or at least weekly	805	74.0	116	61.6	54	67.0	0.6	0.3	1.0	**0.037**	0.8	0.3	1.8	0.553
Are you satisfied with the help and support from close friends?														
Dissatisfied	173	6.8	42	18.5	17	22.1	Ref	—	—	—	Ref	—	—	—
Satisfied	1011	93.2	157	81.5	59	77.9	0.3	0.1	0.6	**0.001**	0.3	0.1	0.9	**0.035**
How often do you see any of your neighbors to have a chat or do something with?														
Never, at least monthly, or less often	623	40.9	129	50.5	54	61.3	Ref	—	—	—	Ref	—	—	—
Daily, 2–3 times a week or at least weekly	996	59.1	138	49.5	64	38.7	0.7	0.5	1.0	0.056	0.5	0.2	0.9	**0.024**
Ever felt suicidal or wished to be dead														
No	1492	99.3	234	92.9	83	53.0	Ref	—	—	—	Ref	—	—	—
Yes	6	0.7	14	7.1	31	47.0	10.9	3.3	36.2	**≤001**	267.3	79.4	899.4	**≤001**
Has done something or planned to do something about killing self														
No	1492	100.0	239	100	99	96.8	—	—	—	—	—	—	—	—
Yes	0	0.0	0	0.0	1	3.2	Not estimated due to zero cells							

*Note:* Bold values indicate statistical significance.

## Data Availability

The data that support the findings of this study are available from the corresponding author upon reasonable request.
